# Architecture for microcomb-based GHz-mid-infrared dual-comb spectroscopy

**DOI:** 10.1038/s41467-021-26958-6

**Published:** 2021-11-12

**Authors:** Chengying Bao, Zhiquan Yuan, Lue Wu, Myoung-Gyun Suh, Heming Wang, Qiang Lin, Kerry J. Vahala

**Affiliations:** 1 T. J. Watson Laboratory of Applied Physics, California Institute of Technology, Pasadena, CA 91125 USA; 2 Department of Electrical and Computer Engineering, University of Rochester, Rochester, NY 14627 USA; 3 Present Address: State Key Laboratory of Precision Measurement Technology and Instruments, Department of Precision Instruments, Tsinghua University, Beijing, 100084 China; 4 Present Address: Physics & Informatics Laboratories, NTT Research, Inc. 940 Stewart Dr, Sunnyvale, CA 94085 USA

**Keywords:** Solitons, Solitons, Frequency combs, Imaging and sensing, Mid-infrared photonics

## Abstract

Dual-comb spectroscopy (DCS) offers high sensitivity and wide spectral coverage without the need for bulky spectrometers or mechanical moving parts. And DCS in the mid-infrared (mid-IR) is of keen interest because of inherently strong molecular spectroscopic signatures in these bands. We report GHz-resolution mid-IR DCS of methane and ethane that is derived from counter-propagating (CP) soliton microcombs in combination with interleaved difference frequency generation. Because all four combs required to generate the two mid-IR combs rely upon stability derived from a single high-Q microcavity, the system architecture is both simplified and does not require external frequency locking. Methane and ethane spectra are measured over intervals as short as 0.5 ms, a time scale that can be further reduced using a different CP soliton arrangement. Also, tuning of spectral resolution on demand is demonstrated. Although at an early phase of development, the results are a step towards mid-IR gas sensors with chip-based architectures for chemical threat detection, breath analysis, combustion studies, and outdoor observation of trace gases.

## Abstract (web summary)

Chip-based architectures for mid-infrared gas sensing could enable many applications. In this direction, the authors demonstrate a microcomb-based dual-comb spectroscopy sensor with GHz resolution in the mid-IR band, with stability completely determined by a single high-Q microresonator.

## Introduction

Dual-comb spectroscopy (DCS) works by mapping an optical comb of frequencies into radio-frequencies by multi-heterodyne beat with a second comb having a slightly different repetition rate. Because the two combs sample absorption spectra with a resolution set by their line spacing (or repetition rate), analysis of the corresponding comb of radio frequencies reveal these spectra in a multiplexed fashion without the use of scanning gratings or interferometers^1–3^. Comb generation in the mid-infrared (mid-IR) has traditionally used methods that rely upon mode-locked pulse generation, including difference-frequency-generation (DFG), optical parametric oscillation, and supercontinuum generation^3,4^; and there is considerable progress using such systems for mid-IR DCS^5–14^. More recently, mid-IR comb generation by DFG using electro-optic frequency combs (EO-comb) has also been demonstrated^15,16^. In contrast to conventional mode-locking, this approach offers rate tunability to the X-band range (8−12 GHz) and higher^16^; and because DCS systems can trade-off spectral resolution for higher acquisition rates, such higher rates can be useful for the study of dynamics^17^. With the advent of thin-film lithium niobate technology, EO-combs have potential for chip-integration^18^. Indeed, on-chip lithium niobate microcavity-based EO-combs have been used for DCS in the near-IR^19^.

Also offering high repetition rates and chip integration are soliton microcombs^20,21^. On account of their compact size, these devices operate readily in the X to millimetre-wave bands. Microcomb-based DCS has been reported at rates of 22 and 450 GHz in the near-IR^22,23^ and 127 GHz in the mid-IR^5^. And while offering extremely short acquisition times, these rates are too high for spectroscopy of many species, leading to spectral under-sampling of gas samples. For balance between acquisition rate and spectral resolution, DCS at GHz rates is considered to be relatively optimal for sensing of ambient gases (with linewidths narrower than 10s of GHz)^24–26^. Special efforts have been directed to reduce near-IR microcomb rates to the single-digit GHz range^27,28^, but these require very high Q resonators to reduce increased threshold pumping power associated with larger mode volumes. Thermal tuning of large spacing microcombs has also been used to improve the resolution for near-IR DCS at the expense of measurement speed^29^. Aside from microcombs, an on-chip III−V laser frequency comb with a line spacing of 1 GHz (together with an EO-comb) has also been used for near-IR DCS^30^. Nonetheless, mid-IR DCS with GHz resolution remains quite challenging for chip-based devices, including quantum cascaded laser frequency combs^31,32^.

Here, we report microcomb-based DCS with GHz resolution in the mid-IR band. The two GHz-rate mid-IR combs are generated by interleaved difference-frequency-generation (iDFG)^33^ applied to four near-IR combs. These four combs are linked to counter-propagating (CP) solitons^34^ formed within a single microcavity. The frequency stability of the resulting mid-IR DCS spectra is high on account of this simplified architecture in combination with the high mutual coherence of the CP solitons. DCS measurements of methane and ethane near 3.3 μm are performed. Normalized precision as high as 1.0 ppm ⋅ m$\cdot \sqrt{{{{{{{{\rm{s}}}}}}}}}$ is demonstrated.

## Results

### Architecture of the DCS system

**Fig. 1:**
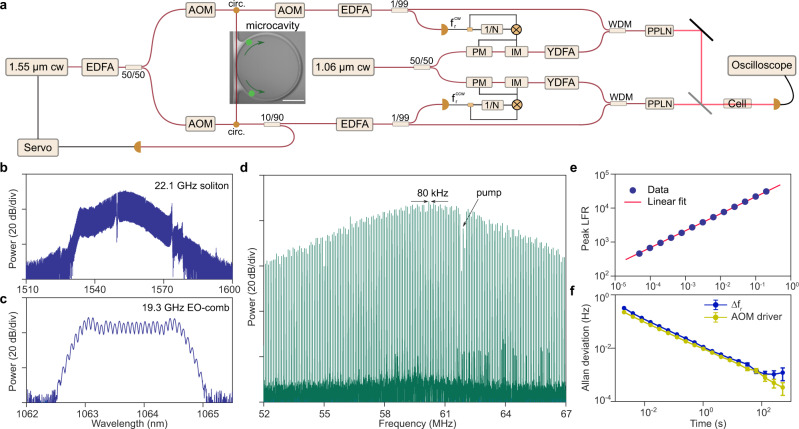
Experimental setup of the GHz-mid-IR DCS system. **a** Counter-propagating (CP) solitons at 1.55 μm are generated in a silica microcavity to provide two of four comb signals. These solitons are photo-detected and the resulting signals are processed to create the two other comb signals by electro-optic modulation at 1.06 μm. These near-IR combs are combined in pairs to pump PPLN crystals for generation of GHz line spacing mid-IR combs by interleaved difference frequency generation. These mid-IR comb sources pass through a gas cell and are detected for dual-comb spectroscopy. Fiber Bragg grating filters used to filter pump waves in the soliton microcomb spectra are omitted in the figure.  ${f}_{\,{{\mbox{r}}}}^{{{\mbox{cw}}}\,}$ (${f}_{\,{{\mbox{r}}}}^{{{\mbox{ccw}}}\,}$) corresponds to the cw (ccw) soliton repetition rates. AOM: acousto-optical modulator, circ: circulator, PM: phase modulator, IM: intensity modulator, EDFA: erbium-doped fibre amplifier, YDFA: ytterbium-doped fibre amplifier, WDM: wavelength division multiplexer, PPLN: periodically poled Lithium Niobate. Scale bar: 1 mm. **b** Optical spectrum of 1.55 μm soliton comb. **c** Optical spectrum of 1.06 μm EO-comb. **d** Multi-heterodyne beat between two CP soliton microcombs (repetition rate difference, Δ*f*_r_, is 80 kHz). The beat note produced by the counter-pumps is identified. One of the microcombs is shifted by 55 MHz using the AOM placed after the cavity (see diagram in panel **a**). **e** Peak LFR of the comb lines in panel (**d**) as a function of averaging time *τ*. The solid line is a fit of the $\sqrt{\tau }$ trend. **f** Measured Allan deviation of Δ*f*_r_ is close to the stability of the AOM driver. The frequency of the AOM driver (a radio frequency function generator) was set to be Δ*f*_r_ in this measurement. The error bar corresponds to the standard deviation of the Allan deviation.

The experimental setup is illustrated in Fig. 1a. It shows two 3.3 μm frequency combs generated in upper and lower branches of the optical train, followed by combining (far right in the figure) for input to the test gas cell. In accordance with the DCS procedure as described elsewhere^2^ the two combs are photodetected after passage through the gas cell, and this multi-heterodyne process creates a radio-frequency spectrum that contains the mid-IR absorption spectrum of the gas. The spectrum is obtained by fast Fourier transform (FFT) of the time-domain interferogram signal of the dual combs. The gas cell (Wavelength Reference) has a length of 5 cm and contains ~2% methane (CH_4_) and ~0.5% ethane (C_2_H_6_) buffered by nitrogen to a total pressure of 760 Torr (parameters can have ±5% uncertainty). Such a methane concentration is equivalent to about 1 ppm in an ambient environment when passing the comb light through a 1 km open path for field measurements.

Each mid-IR comb is generated by iDFG in a PPLN crystal (4 cm long, NTT Electronics) of two near-IR combs: a soliton microcomb at 1.55 μm (Fig. 1b) and an EO-comb (Fig. 1c) at 1.06 μm. Counter-pumped clockwise (cw) and counter-clockwise (ccw) solitons formed in a single silica resonator^35^ are input to upper and lower branches of the optical train. On account of the silica Raman response, the soliton repetition rates (${f}_{\,{{\mbox{r}}}}^{{{\mbox{cw}}}\,}$ or ${f}_{\,{{\mbox{r}}}}^{{{\mbox{ccw}}}\,}$) can be independently fine-controlled by two acousto-optical modulators (AOMs) placed before the resonator^34^. Their approximate repetition rate is 22 GHz. Another AOM after the microcavity, driven by a fixed 55 MHz signal, is used to shift the frequencies of one of the microcombs so as to avoid spectral aliasing upon multi-heterodyne beating of the two combs in both the near-IR and the mid-IR. Each EO-comb drive frequency is derived from a corresponding photo-detected soliton repetition frequency and set to be $(N-1){f}_{\,{{\mbox{r}}}}^{{{\mbox{cw(ccw)}}}\,}/N$ (*N* is an integer). This results in interleaving of the near-IR combs and densifies the mid-IR comb line spacing to 22 GHz/*N* as described elsewhere^33^. 2.8 and 1.4 GHz mid-IR line spacings are demonstrated, corresponding to *N* = 8 or 16.

For high precision measurements, the two mid-IR comb spectra must have excellent relative frequency stability. Several features of the current system architecture ensure this result while also reducing the system complexity. First, the upper and lower optical trains share common near-IR continuous-wave pumping lasers. These pumps or their AOM-shifted replicas become comb lines in each of the four near-IR combs. EO-combs, therefore, have identical centre frequencies, while soliton combs have offset frequencies that are related by the difference in the AOM frequency shifts (Δ*ν*_P_) applied to the soliton pumps. Second, by tuning the relative counter-pumping frequency Δ*ν*_P_, the repetition rates of the two microcombs (${f}_{\,{{\mbox{r}}}}^{{{\mbox{cw}}}\,}$ and ${f}_{\,{{\mbox{r}}}}^{{{\mbox{ccw}}}\,}$) become phase-locked such that Δ*f*_r_ = Δ*ν*_P_/*M* (${{\Delta }}{f}_{{{{{{{{\rm{r}}}}}}}}}={f}_{\,{{\mbox{r}}}}^{{{\mbox{cw}}}}-{f}_{{{\mbox{r}}}}^{{{\mbox{ccw}}}\,}$ and *M* is an integer)^34^. Because the EO-comb rates are derived from the soliton comb rates, all four combs, despite having different repetition rates, have their rates phase-locked. This feature in combination with the common optical pumps means that the two mid-IR combs have an offset frequency noise equal to the fluctuations in the difference frequency of the 1.55 and 1.06 μm pumps as their primary source of frequency instability. Significantly, however, this is a common-mode fluctuation to the mid-IR combs and will therefore cancel out in the multi-heterodyne DCS detection process. As a result, the frequency stability of the mid-IR comb interferogram is extremely high, being primarily determined by the relative stability of the two CP solitons. This stability is accomplished without the need for frequency locking procedures, because of the above-mentioned features of the system architecture. Moreover, this architecture eliminates the need for bulk microwave oscillators required for EO-combs.

To illustrate the frequency stability that is possible using this architecture, a portion of the Fourier transform of the measured dual-soliton interferogram (measured on a balanced receiver over 200 ms) is shown in Fig. 1d. Defining the line-to-floor ratio (LFR) as the square root of the ratio of signal power to the average noise floor power (see “Methods”), Fig. 1e shows that the highest LFR of the radio-frequency comb scales as 6.3$\times\! 1{0}^{4}\sqrt{\tau }/\sqrt{{{{{{{{\rm{s}}}}}}}}}$ (*τ* is the measurement time). This value gives a measure of dynamic range available for absorption measurement. The mutual stability of the two microwave signals generated by photodetecting the soliton streams was also tested by mixing ${f}_{\,{{\mbox{r}}}}^{{{\mbox{cw}}}\,}$ and ${f}_{\,{{\mbox{r}}}}^{{{\mbox{ccw}}}\,}$. The measured Allan deviation of their difference frequency Δ*f*_r_ (Fig. 1f) shows that the two soliton microwave rates reach a relative frequency fluctuation less than 1 Hz at around a millisecond of averaging time. Then, the stability further improves to about 1 mHz at 100 s. This Allan deviation is found to be close to that of the frequency fluctuation of the AOM driver when setting its output frequency close to Δ*f*_r_ (see Fig. 1f).

### Characterization of the DCS system

**Fig. 2:**
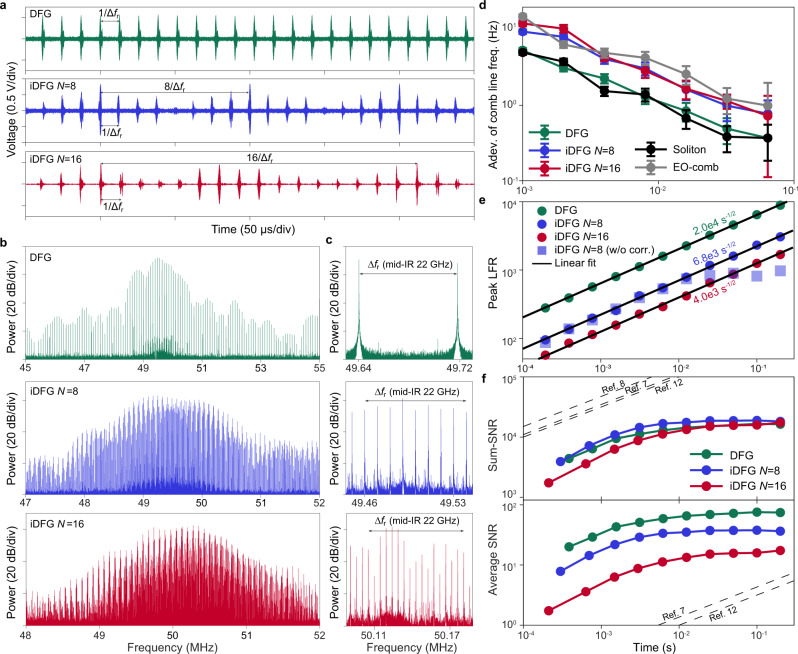
Interferograms and multi-heterodyne spectra of the iDFG densified mid-IR combs. **a** Interferogram of the mid-IR combs using DFG and iDFG. The interferograms for the iDFG combs repeat at a rate of Δ*f*_r_/*N*. **b** Dual-comb spectra formed by fast Fourier transform of the measured interferograms. The radio frequency combs are spectrally densified when using iDFG. **c** Zoom-in of the radio-frequency combs in panel (**b**). A 22 GHz scale giving the corresponding optical bandwidth in the mid-IR is provided. **d** Allan deviation of the frequency of a single line measured at the centre of the multi-heterodyne spectra plotted versus the measurement time. The generation mechanism is indicated in the legend. The error bar corresponds to the standard deviation of the Allan deviation. **e** Plot of the LFR versus measurement time *τ* for strongest spectral line using conventional and interleaved DFG. The solid lines are linear fits ($\sqrt{\tau }$ trend in the log−log plot). All circular points are digitally corrected. Squares points show the uncorrected results for *N* = 8. **f** Plot of the sum- and average SNR of our mid-IR DCS system versus the measurement time *τ*. Black dashed lines are SNR for reported mid-IR DCS systems.

The transfer of mutual coherence of the CP solitons to the mid-IR is verified in Fig. 2a where measured interferograms of the mid-IR combs are displayed. The mid-IR interferograms are collected by a fast photodetector (600 MHz bandwidth, PVI-4TE-4, Vigo System SA), and the optical power is around 60 μW (Supplementary Note 1) to avoid detector nonlinearity. For comparison, interferograms are shown using conventional DFG (EO-comb drives turned off for soliton mixing with the 1.06 *μ*m continuous-wave laser) as well as iDFG with *N* = 8 and *N* = 16. In the DFG case, interferogram pulses repeat at the rate of Δ*f*_r_, while in the iDFG case the pulses repeat at the rate of Δ*f*_r_/*N*. Note that even for the iDFG case, there are pulses appearing at the rate Δ*f*_r_ and it is the envelope modulation of these pulses that reflect the interleaving process. This non-ideal behaviour is mainly the result of three effects that could be corrected in the future. First, the EO-comb pulses themselves were not fully compressed (i.e., not tranform limited), because of the lack of two dispersion control systems. The dual-EO-comb interferogram suggests the extinction ratio of the EO-pulses is about 8 dB. Second, based on the group refractive index difference and length of the commercial PPLN crystal, the temporal walk-off between two near-IR pulses is estimated to be up to ~4 ps. Third, the PPLN phase-matching bandwidth for this long crystal is quite narrow (<300 GHz) at 1.5 *μ*m and leads to an effectively wider soliton pulse width. A criterion for minimal residual pulses in iDFG can be given as,

1 $${t}_{{{{{{{{\rm{s}}}}}}}}}+{t}_{{{{{{{{\rm{EO}}}}}}}}}+| {{\Delta }}{t}_{{{{{{{{\rm{wkf}}}}}}}}}| < {T}_{{{{{{{{\rm{s}}}}}}}}}/N,$$

where *t*_s_ (*t*_EO_) is the effective width of the soliton (EO) pulse, Δ*t*_wkf_ is the walk-off between the near-IR pulses in the crystal, and *T*_s_ is the 1.55 μm soliton period. A detailed discussion of the residual pulses is given in the Supplementary Note 4. The use of fully compressed EO-combs in combination with a more optimal PPLN would eliminate the residual RF pulses in the interferograms. In such an optimized arrangement only one RF pulse would appear in the time period of *N*/Δ*f*_r_. This would also eliminate spectral envelope modulation in the interferogram FFT as noted below.

As an aside, Δ*f*_r_ was tuned slightly in the measurements and, as a result, the RF pulses are not aligned between the three cases in Fig. 2a. Furthermore, the PPLN temperature (and thus the phase-matching condition) was also tuned in experiments so that the RF pulses exhibit different envelope modulation for the iDFG *N* = 8 and *N* = 16 cases.

FFT of the interferograms yields the radio-frequency combs shown in Fig. 2b. Digital correction described in the “Methods” was used here to compensate fluctuations induced by fibres connecting comb generation and gas cell spectroscopy setups, which resided in different laboratories (see discussion relating to Fig. 2e in Supplementary Note 2). Co-location of the setups to a single table (or ultimately integration of the components) should avoid this fluctuation and simplify data processing. In the spectra, the conventional DFG case has a line spacing of Δ*f*_r_, while the iDFG cases have a line spacing narrowed to Δ*f*_r_/*N*. Spectral envelope modulation appearing for *N* = 8 and *N* = 16 (compare to conventional DFG spectrum) results from the non-ideal residual pulses discussed in Fig. 2a. A zoom-in of the RF comb spectra in Fig. 2c shows the densification of the lines.

As noted above, the full optical microcomb bandwidth was not available for mid-IR comb generation due to the limited phase-matching bandwidth of the PPLN crystals. This limitation can be observed in the DFG interferogram spectrum in Fig. 2b, where the comb line intensity decreases rapidly from the spectrum centre. To somewhat reduce this limitation in the current measurements, the temperature of the two PPLN crystals was set to be slightly different so as to enlarge the usable bandwidth in the mid-IR.

To measure the absolute frequency stability of the dual-comb interferogram spectra, the Allan deviation of a single multi-heterodyne beat frequency is calculated in Fig. 2d for mid-IR, CP and EO generated spectra. Using conventional DFG the frequency stability is comparable to that of the near-IR CP solitons. Here, the stability is better than 1 Hz within 100 ms averaging time as a result of stability linked to the single microcavity. A slight degradation is observed for the iDFG *N* = 8 and iDFG *N* = 16 cases, which may result from additional noise contributed by the EO-combs. This is substantiated in Fig. 2d by Allan deviation measurement of a single frequency within a multi-heterodyne spectrum produced using only two 1.06 μm EO combs.

To further confirm the mutual phase coherence in the mid-IR, the LFR of the mid-IR interferogram spectra (calculated using the strongest spectral peak) is analyzed in Fig. 2e. In all three cases (DFG, iDFG *N* = 8, and iDFG *N* = 16), the LFR shows a $\sqrt{\tau }$ trend (after digital correction), and scales as 2.0$\times 1{0}^{4}\sqrt{\tau }/\sqrt{{{{{{{{\rm{s}}}}}}}}}$, 6.8$\times 1{0}^{3}\sqrt{\tau }/\sqrt{{{{{{{{\rm{s}}}}}}}}}$, and 4.0$\times 1{0}^{3}\sqrt{\tau }/\sqrt{{{{{{{{\rm{s}}}}}}}}}$, respectively. Data obtained without digital correction (squares) for iDFG *N* = 8 are also presented and show that the mutual coherence is preserved up to 10 ms until the aforementioned fibre fluctuations cause deterioration (see Supplementary Note 2). The high LFR can enable high dynamic range spectroscopic measurements.

To further characterize the DCS system, the signal-to-noise ratio (SNR: square root of the ratio of the signal to the standard-deviation of the fluctuations)^2,7–9,15^ of lines in the FFT of the interferogram is calculated (see “Methods”). These values are then used to compute the sum-SNR (upper panel of Fig. 2f) and average SNR (lower panel of Fig. 2f) versus integration time over lines within 40 dB of the strongest line^2^. The sum-SNR initially increases as $\sqrt{\tau }$ within 2 ms, but then saturates at longer averaging times. For comparison, the sum-SNRs for other reported mid-IR DCS systems are plotted as dashed lines. Considering the good frequency stability of the present system, the relatively low sum-SNR is likely limited by the amplitude noise of the mid-IR combs. It is possible that this could result from the use of fibre-taper optical coupling to the resonator, which can introduce a mechanism for environmental noise to impact coupling. The use of a fully integrated microcomb would avoid this problem. Moreover, the use of a reference mid-IR photodetector would enable monitoring of power fluctuations^22^ and could help to boost the sum-SNR. Although the sum-SNR is relatively low, the average SNR of the spectrum is relatively high and enables a fast measurement. This results from fewer usable lines in the current system compared with the fibre-based mid-IR systems.

### Mid-IR DCS of methane and ethane

**Fig. 3:**
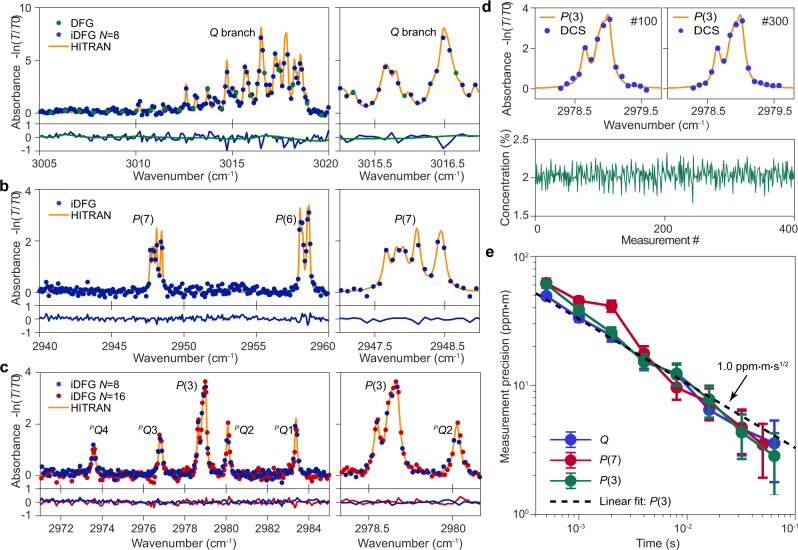
Dual-comb spectroscopy (DCS) of methane and ethane mixture using iDFG mid-IR combs. **a** Absorbance spectrum for the methane *ν*_3_ band *Q* branch using DFG and iDFG (*N* = 8). iDFG improves the spectral resolution compared to the 22 GHz mid-IR generated by conventional DFG. HITRAN data are indicated by the yellow line. The right panel (and in **b, c**) is a zoom-in of absorption features. The `observed-HITRAN' residuals are shown in the lower panel (also in **b, c**). **b** DCS absorbance spectrum of the methane *P*(6) and *P*(7) branches in the *ν*_3_ band measured using iDFG with *N* = 8. **c** Absorbance spectrum of the methane *P*(3) branch in the *ν*_3_ band together with the ethane rovibrational transitions in the *ν*_7_ band. Since ethane has a narrower absorption linewidth, iDFG with *N* = 16 was also used to further improve the spectral resolution. **d** Top panels are DCS spectra of the methane *P*(3) branch (iDFG *N* = 8), each measured over 0.5 ms duration within a 200 ms measurement window. 100th and 300th spectra of 400 total are displayed. Fitting the spectrum to the HITRAN database yields the methane concentration. The lower panel plots the concentration for the 400 time slots. **e** Normalized measurement precision of methane concentration evaluated by Allan deviation using different rovibrational transitions belonging to different branches with iDFG *N* = 8. The error bar corresponds to the standard deviation of the Allan deviation. The Allan deviation of the measured concentration (e.g., lower panel in **d**) is calculated and normalized to a 1 m optical path. The precision scales nearly as $1/\sqrt{\tau }$ and the dashed line is the corresponding linear fit using a $1/\sqrt{\tau }$ trend line (log−log) for the *P*(3) branch. A precision of 1.0 ppm ⋅ m$\cdot \sqrt{{{{{{{{\rm{s}}}}}}}}}$ is fitted.

The mid-IR DCS system was used to measure the absorbance spectra of a mixture of methane and ethane gas. The dual-comb spectrum with the gas cell inserted (*T*) was first measured and then normalized by the reference spectrum measured without the gas cell (*T*_0_). The absorbance is then calculated as $-{{{{{{\mathrm{ln}}}}}}}\,(T/{T}_{0})$. The wavelength of the 1.06 μm laser (an external cavity diode laser) was tuned to access rovibrational transitions belonging to different branches in the *ν*_3_ band of methane^36^. The *Q*-branch of methane around 3015 cm^−1^ was first measured. The absorbance spectra measured by DFG and iDFG *N* = 8 DCS are presented in Fig. 3a. We compare the measured spectra to the HITRAN database using the gas cell information given above (the absolute frequency offset was used as a free parameter for the best fit). While both absorbance spectra are in a good agreement with the HITRAN database^37^ the DFG spectrum undersamples the methane spectral features due to its relatively wide 22 GHz comb line spacing, and only three data points (green points) appear for the zoom-in spectrum in Fig. 3a. On the other hand, this spectral undersampling is avoided by using iDFG DCS with a reduced comb line spacing of 2.8 GHz corresponding to iDFG with *N* = 8. The residuals between the measured spectrum and HITRAN database are plotted in the lower panel. The non-negligible residuals reflect the relatively low sum-SNR shown in Fig. 2f. Additional data obtained for the *P*-branch of methane (e.g., *P*(3), *P*(6), and *P*(7)) are presented in Fig. 3b, c. The measured spectra are also in good agreement with the HITRAN database. The ethane absorption spectrum in the *ν*_7_ band^38^ was also measured in Fig. 3c. Such an ability to measure the methane and ethane simultaneously is important to distinguish if the methane emission comes from gas wells^39^.

A feature of iDFG DCS is that the spectral resolution can be adjusted by changing the division ratio *N*^33^. For instance, the full-width at half-maximum (FWHM) of methane *P*(3) to *P*(7) transition groups in the *ν*_3_ band are within 10−26 GHz, while the FWHM of ethane ^*P*^*Q*_1_ to ^*P*^*Q*_4_ transitions in the *ν*_7_ band are within 4.2−6.9 GHz according to the HITRAN database. Improved resolution of the ethane absorbance via iDFG DCS is shown as the red dots in Fig. 3c. Here, a finer resolution of 1.4 GHz is achieved by selecting *N* = 16. In principle, the resolution of the DCS system could be adjusted in steps from GHz to 22 GHz, making it possible to optimize resolution and SNR depending upon the characteristics of the gas sample.

The GHz DCS system also enabled fast and precise measurement of the absorbance spectrum. Measurement precision is evaluated using the Allan deviation of the measured methane concentration in the 5 cm cell. 200 ms interferograms were separated into 400 slots and the methane concentration was calculated in each resulting 0.5 ms slot (corresponding to about 5 interferogram periods for iDFG *N* = 8). Figure 3d details the evaluation process for the *P*(3) branch measurement with iDFG *N* = 8. The top panels are representative spectra from two 0.5 ms time slots (numbers 100 and 300) without any digital correction as mutual coherence is preserved. They illustrate fast acquisition of the methane absorption spectrum, which can result from the relative high average SNR achieved in short time. Fitting each absorbance spectrum to the HITRAN database yields the measured methane concentration in each time slot (lower panel of Fig. 3d). Since about 20~30 comb lines in the absorption spectra are used to fit for the concentration, the residuals between the observation and HITRAN are found to not significantly degrade the measurement precision. This measured concentration sequence was then used to calculate the Allan deviation of the measured methane concentration, which was further normalized by the gas cell length to derive the normalized measurement precision in Fig. 3e (all for the iDFG *N* = 8 case). The Allan deviation (*P*(3) branch measurement) reaches a precision of ~2.8 ppm ⋅ m within 64 ms. In fitting the Allan deviation of the *P*(3) branch measurement to a $1/\sqrt{\tau }$ trend line, a normalized measurement precision of 1.0 ppm ⋅ m$\cdot \sqrt{{{{{{{{\rm{s}}}}}}}}}$ can be obtained. Measurements of rovibrational transitions in other branches produce similar results. This sub-ms measurement time may make this system suitable for studies of transient events in combustion^17^.

The measurement acquisition times of 200 ms for Fig. 3a, c and 100 ms for Fig. 3b are shorter than fibre-comb-based mid-IR DCS systems, which generally require an acquisition time longer than tens of seconds^8,11,12^ (usually those systems have a much larger comb line number). Even shorter acquisition times should be possible that are comparable to EO-comb-based systems, where mid-IR DCS has also been demonstrated^15^. To attain a shorter acquisition time, CP solitons with a larger Δ*f*_r_ generated on distinct mode families could be utilized^40^.

## Discussion

**Fig. 4:**
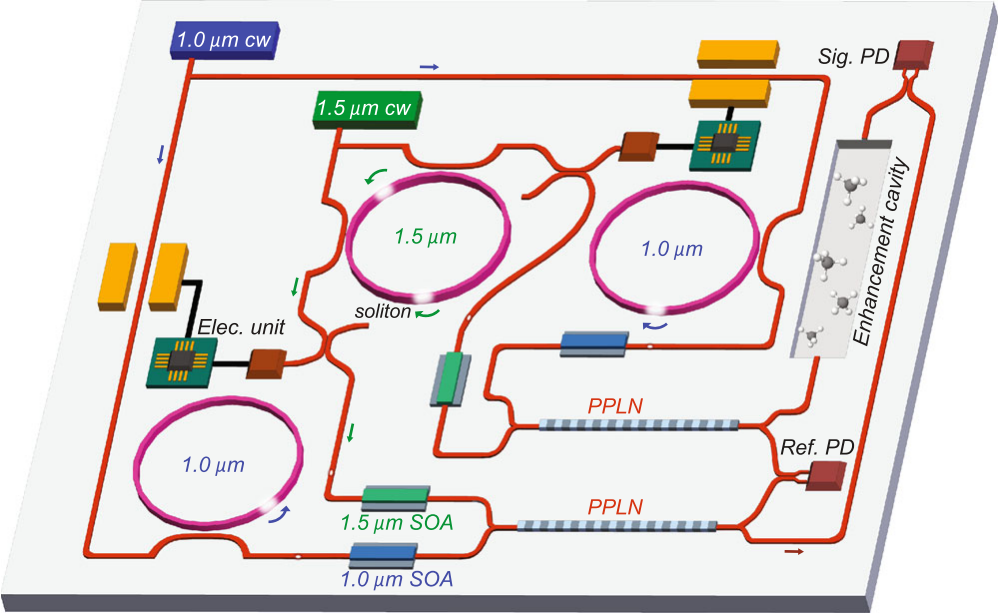
A conceptualization of a monolithically integrated mid-IR DCS sensor. Two 1.0 μm solitons phase-locked to the 1.5 μm CP solitons are used for iDFG in on-chip PPLN waveguides. SOA: semiconductor optical amplifier.

In this work, microcomb-based DCS in the mid-IR with GHz resolution has been demonstrated. This represents a 100-fold improvement in spectral resolution compared with previous mid-IR microcomb DCS. Mutual coherence of near-IR CP solitons enables precise methane absorption measurements reaching a normalized precision of 1.0 ppm ⋅ m$\cdot \sqrt{{{{{{{{\rm{s}}}}}}}}}$. While the demonstrated system still relies upon fibre optics, further integration of the system on a photonic chip is feasible. Along this direction, both near-IR combs as well as the DFG crystal can potentially be monolithically or hybridly integrated in the future. In particular, thin-film LiNbO_3_ microcavities and waveguides have been demonstrated to function as soliton microcomb generators^41^, EO modulators^18^ and as PPLN wavelength converters^42,43^. A conceptualization of such a mid-IR DCS system is shown in Fig. 4 wherein the EO-combs are replaced by two 1 μm soliton microcombs whose repetition rates are locked to the desired frequencies by injection locking^44^. Using solitons as opposed to EO-combs can offer higher peak power and therefore more efficient iDFG. However, their application for iDFG may also limit the rate reconfigurability shown in Fig. 3c. EO-combs could also be integrated to retain this reconfigurability. Also, cavity-enhanced DCS^45^ can be used to further increase the measurement sensitivity for the proposed mid-IR chip-based sensor (folding of the light path will be needed to reduce the footprint of the GHz enhancement cavity).

Moreover, rapid progress in the overall subject of microcomb systems should ultimately address gaps in the current platform required for complete integration^46–48^. Such photonic integration can bring opportunities to improve the system performance. For example, the tight mode confinement of on-chip PPLN waveguides can allow dispersion engineering to achieve more optimal phase-matching for increased broadband spectral coverage in the mid-IR^49^. Tight mode confinement can also increase the conversion efficiency. Ultimately, integration of the DCS system would enable miniature (potentially hand-held) gas sensors with high-resolution capability for the detection of gases in a cluttered environment. Such a robust sensor technology would be suitable for widespread spectroscopy applications in laboratories and field measurements.

## Methods

### Signal-to-noise ratio (SNR) of the DCS system

The SNR is defined as the square root of the ratio of the spectral line power *P*_*n*_ (for the *n*-th spectral component of the FFT of the interferogram) to the average of the standard-variance of uncorrelated fluctuations in all spectral lines. This definition is widely used to evaluate DCS systems^2,8,9,15^. In practice, a smooth baseline (spectral envelope) is used to fit the multi-heterodyne spectrum and the residual fluctuations of spectral lines relative to this baseline provide a series of standard variance values that are combined to compute the average standard-variation, the square root of which provides a standard deviation (*σ*). This gives the SNR of the *n*-th spectral line as follows:

2 $${{{{{{{\rm{SN{R}}}}}}}_{n}}}(\tau )\equiv \sqrt{\frac{{P}_{n}(\tau )}{\sigma (\tau )}}$$

where *P*_*n*_ is the electrical power in the *n*-th spectral line, and both this power and the standard deviation are measured over an integration time *τ*. However, in the present case there is a complication introduced by the residual pulses in the interferogram. As discussed in the main text, these create a complex spectral envelope that is difficult to fit to a spectral envelope. Therefore, as an alternative, we use the FFT spectrum taken when the interferogram FFT is performed for 200 ms, but over a different interval of time. This reference choice is consistent with the way we calculated DCS spectra in Fig. 3.

The sum-SNR presented in Fig. 2e is derived from the above SNR_*n*_ by summing over *n* where *n* is restricted to those lines of which the minimum power is 40 dB lower than the strongest comb line. The average SNR is calculated as the sum-SNR divided by the comb line number (again, taken as comb lines for which the minimum power is 40 dB smaller than the strongest comb line). It is worth noting that the sum-SNR can further increase after 2 ms if the comb spectrum of the same 200 ms interferogram is used as the reference to calculate SNR versus averaging time (see Supplementary Note 3). This suggests the sum-SNR can be improved when a reference mid-IR photodetector is used to monitor the intensity fluctuations.

### Line-to-floor ratio (LFR)

The ‘line’ here refers to the spectral line signal in the multi-heterodyne beat signal produced by Fourier transform of the interferogram. The ‘floor’ is the noise floor measured mid-span between the line and its nearest neighbour. Both signal and noise floor are determined for a range of FFT integration times *τ* (equivalent to varying bandwidth or to varying the interferogram time length). We define the LFR measured with integrated time *τ* of a certain comb line *n* as the square root of the ratio of the comb line power (*I*_*n*_) to the noise floor 〈*I*_F_(*τ*)〉.

3 $${{{{{{{\rm{LF{R}}}}}}}_{n}}}(\tau )\equiv \sqrt{\frac{{I}_{{{{n}}}}(\tau )}{\langle {I}_{{{{{{{{\rm{F}}}}}}}}}(\tau )\rangle }}.$$

The peak LFR is the LFR of the comb line with the highest signal power.

### Dual-comb signal processing

In principle, the iDFG mid-IR combs should have very high mutual coherence, limited only by AOM radio-frequency drive sources. However, in the current arrangement, separate optical fibres convey the CP solitons from one laboratory to another in order to accommodate the equipment required in the experiment. This fibre transport is observed to cause technical fluctuations in Δ*f*_r_ and Δ*f*_0_ (offset frequency difference between the two combs). To correct these fluctuations, the dual-comb data are processed using the computational coherent averaging algorithm (CoCoA)^50^. First, Δ*f*_r_/*N* fluctuations are tracked from FFT of the time domain signal envelop (derived by Hilbert transform of the interferogram). In this way, the Δ*f*_0_ influence can be eliminated. The tracked Δ*f*_r_/*N* later are corrected by resampling onto a new non-uniform time axis. Then, Δ*f*_0_ fluctuations are tracked according to a recursion algorithm^50,51^. This fluctuation was corrected by subtracting the corresponding phase fluctuations of the interferograms. In the future, co-location of all hardware on a common optical table (or integration of the system onto a chip) should avoid these fluctuations. Indeed, the dual-comb spectrum presented in Fig. 1c, which was measured on the same table with the CP soliton setup, was obtained by direct Fourier transform without digital correction.

### Allan deviation of the absorption precision

The Allan deviation is calculated as

4 $${\sigma }_{\alpha }(\tau )\equiv \sqrt{\frac{1}{2(m-1)}\mathop{\sum }\limits_{i=1}^{m-1}{\left({\alpha }_{i+1}-{\alpha }_{i}\right)}^{2}}$$

where *α* is the measured methane concentration in different time slots, *m* is the sample number of *α*, and *τ* is the averaging time. The 5 cm length of the cell is normalized to yield the *y*-axis in Fig. 3e. The Allan deviation in Figs. 1f and 2d is calculated in a similar way.

## Supplementary information

Supplementary Information

Description of Additional Supplementary Files

Supplementary Movie 1

Supplementary Movie 2

Supplementary Movie 3

Supplementary Movie 4

## Data Availability

The data that supports the plots within this paper and other findings of this study are available from the corresponding author upon reasonable request.

## Code availability

The code that supports dual-comb signal processing and other findings of this study are available from the corresponding author upon reasonable request.
