# Supplementary material for: Architecture for microcomb-based GHz-mid-infrared dual-comb spectroscopy
Source: Nat Commun. 2021 Nov 12;12:6573. doi: 10.1038/s41467-021-26958-6 (PMC8589843; doi:10.1038/s41467-021-26958-6)
Supplement: Supplementary file 1 — Supplementary Information [file 41467_2021_26958_MOESM1_ESM.pdf]

Supplementary Information for "Architecture for microcomb-based GHz-mid-infrared dual-comb spectroscopy"

Chengying Bao[1,†,‡], Zhiquan Yuan[1,†], Lue Wu[1], Myoung-Gyun Suh[1,§], Heming Wang[1], Qiang Lin[2] and Kerry J. Vahala[1,*]
[1]T. J. Watson Laboratory of Applied Physics, California Institute of Technology, Pasadena, California 91125, USA.
[2]Department of Electrical and Computer Engineering, University of Rochester, Rochester, NY 14627, USA.
[‡]Present address: State Key Laboratory of Precision Measurement Technology and Instruments, Department of Precision Instruments, Tsinghua University, Beijing 100084, China.
[§]Present address: Physics & Informatics Laboratories, NTT Research, Inc. 940 Stewart Dr, Sunnyvale, California 94085, USA.
[†]These authors contributed equally to this work.
[*]Corresponding author: vahala@caltech.edu

Supplementary Note 1. Mid-IR comb power and PPLN conversion efficiency

The iDFG efficiency was measured to be around 2% /W. In our experiment, the optical power sent into a PPLN crystal at 1.55 μm (1.06 μm) is 80 mW (100 mW). Thus, the mid-IR comb was estimated to have a power of about 160 μW. Due to free space loss and non-optimal spatial overlap at the mid-IR photodetector, the detected optical signal power at 3.3 μm was estimated to be around 60 μW.

Supplementary Note 2. Impact on LFR of conveying near-IR combs to an adjacent laboratory

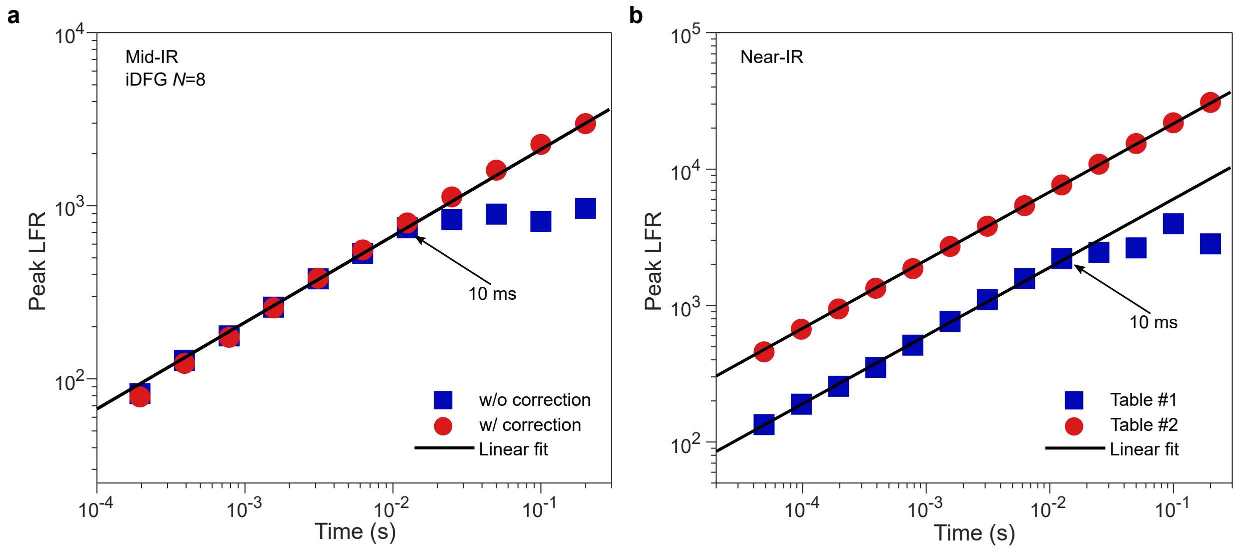

Supplementay Fig. 1: Peak LFR versus averaging time. a DCS LFR in the mid-IR both with and without digital correction using iDFG N=8. The solid line is linear fits (log-log) using $\sqrt{\tau}$ trend line. **b** LFR in the near-IR for dual-soliton multi-heterodyne beat signal (CP solitons used). Soliton generation and beat signal detection on a single table (Table 2) and with detection on a separate table (Table 1) are plotted. The solid lines are linear fits (log-log) using $\sqrt{\tau}$ trend lines.

As noted in the main text, the experiment was conducted in two laboratory bays. And, as a result. the near-IR comb signals generated in one bay were conveyed by optical fibre into the adjacent lab bay prior to difference frequency generation. Path length fluctuations associated with the fibre transport introduced a source of fluctuation that was digitally corrected. To characterize the corrected signal, we use the line-to-floor ratio (LFR) that was introduced in the main text and Methods. Supplementary Fig. 1a shows the LFR versus τ with and without digital correction. Both plots scale as $\sqrt{\tau}$ for short time duration (<10 ms). At longer times there is drift in the uncorrected signal that is believed to be produced by the aforementioned path length fluctuations in the optical fibres. To verify this conjecture, the LFR of a line in the near-IR dual-soliton beat spectrum is also characterized (Supplementary Fig. 1b). When the measurement is performed entirely on the same optical table (Table #2), the LFR scales as $\sqrt{\tau}$ throughout the

measurement time (same data as in Fig. 1e in the main text). However, when conveying the CP solitons to a second table (Table #1) by two optical fibres, the LFR no longer scales as $\sqrt{\tau}$ for measurement times >10 ms (consistent with the results in Supplementary Fig. 1a). This indicates that the optical fibres cause the measured drift. Note that two different near-IR photodetectors were used here and the absolute LFRs in Supplementary Fig. 1b are accordingly different.

Supplementary Note 3. Same-interferogram-referenced sum-SNR

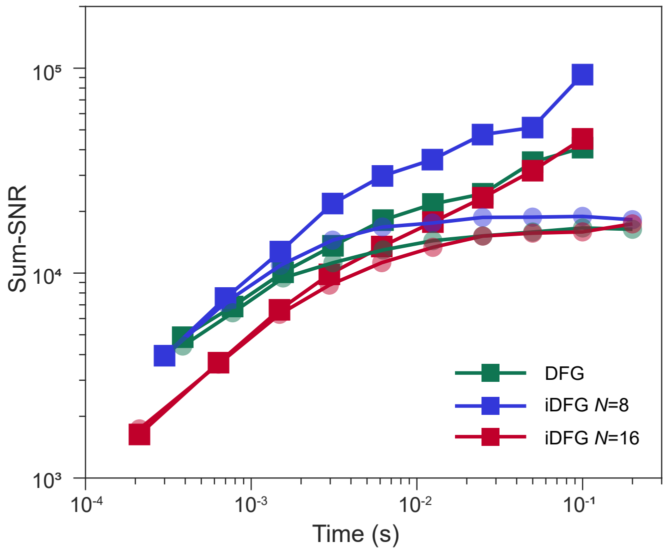

Supplementay Fig. 2: Sum-SNR. Re-production of the upper panel in Fig. 2f of the main text giving the sum-SNR in the mid-IR plotted as a function of measurement time (circles). When the spectrum from the Fourier transform of the 200 ms interferogram used to compute the signal in Fig. 2f is also used as the reference to calculate σ (Eqn. 2 of the main text), then the sum-SNR continues to increase in time (squares).

The sum-SNR from Fig. 2f of the main text is reproduced as the circle points in Supplementary Fig. 2. As noted before, the sum-SNR saturates after 2 ms. A possible explanation of this saturation is that the interferogram used to calculate the signal and the reference interferogram used to calculate σ (Eqn. 2 of the main text) were recorded at different times because of the lack of a second photodetector for use in the reference arm. To test this proposition, we also used the signal interferogram (as opposed to the reference) to calculate σ. The results are shown as the square points in Supplementary Fig. 2. In the first 2 ms, the squares almost overlap with the circles. However, the sum-SNR given by the squares further increases after 2 ms. This suggests that by using a second photodetector to simultaneously record the reference interferogram (see Fig. 4 for an example), it should be possible to improve the sum-SNR of the current system.

Supplementary Note 4. Simulation of residual pulse formation in iDFG

In this section, descriptions of four animated simulations of the iDFG process are provided. Each animation illustrates the impact of a process non-ideality on the appearance of residual peaks in iDFG interferogram (see Fig. 2a in main text). As a result of these simulations, it should be possible to eliminate the residual peaks (and their associated impact on spectral modulation of the fast Fourier transform) with straightfoward modifications of the system. In each GIF file, the time domain signal of 1.5 μm soliton (pulse width is assumed to always be 130 fs), 1 μm EO-comb and 3 μm mid-IR comb pulse are plotted in three panels. The carrier frequency in the 1 μm EO-comb is not plotted so as to clearly illustrate the envelope of the sinc function. Descriptions of the non-ideality studied in each GIF file is now given:

Supplementary Movie 1: The full width at half maximum (FWHM) of the EO-comb is swept from 10 ps to 1 ps. All other conditions are ideal: enough phase matching bandwidth, no walk-off between the two near-IR pulses and no initial time delay between two near-IR pulses at the PPLN input. Residual pulses in the mid-IR can be observed, and result from modulations in the 'tail' of the sinc-function.

Supplementary Movie 2: The relative temporal walk-off between soliton and EO comb pulses is swept from 7 ps to 1 ps. Assuming a limited phase matching bandwidth, the pulse width of the EO-comb is 2 ps and there is no

initial delay between two near-IR pulses.

Supplementary Movie 3: The initial delay between soliton and EO-comb pulses is swept from 5 ps to −5 ps. Assuming sufficient phase matching bandwidth, the pulse width of the EO-comb is 2 ps and the walk-off between two near-IR pulses is 4 ps.

Supplementary Movie 4: The initial delay between the soliton and EO-comb pulses is swept from 5 ps to −5 ps. Assuming limited phase matching bandwidth, the walk-off between the two near-IR signals is 4 ps, the EO-comb is not compressed and is phase chirped. This case is close to actual experimental conditions, because, as noted in the main text, we lacked dispersion compensation units to properly compress both of the EO-combs.
