# Supplementary material for: Architecture for microcomb-based GHz-mid-infrared dual-comb spectroscopy
Source: Nat Commun. 2021 Nov 12;12:6573. doi: 10.1038/s41467-021-26958-6 (PMC8589843; doi:10.1038/s41467-021-26958-6)
Supplement: Supplementary file 2 — Description of Additional Supplementary Files [file 41467_2021_26958_MOESM2_ESM.pdf]

Description of Additional Supplementary Files

File name: Supplementary Movie 1

Description: Simulation of residual pulse formation in iDFG. The full width at half maximum (FWHM) of the EO-comb is swept from 10 ps to 1 ps. All other conditions are ideal: enough phase matching bandwidth, no walk-off between the two near-IR pulses and no initial time delay between two near-IR pulses at the PPLN input. Residual pulses in the mid-IR can be observed, and result from modulations in the `tail' of the sincfunction.

File name: Supplementary Movie 2: Simulation of residual pulse formation in iDFG.

Description: The relative temporal walk-off between soliton and EO comb pulses is swept from 7 ps to 1 ps. Assuming a limited phase matching bandwidth, the pulse width of the EO-comb is 2 ps and there is no initial delay between two near-IR pulses.

File name: Supplementary Movie 3

Description: Simulation of residual pulse formation in iDFG. The initial delay between soliton and EO-comb pulses is swept from 5 ps to -5 ps. Assuming sufficient phase matching bandwidth, the pulse width of the EO-comb is 2 ps and the walk-off between two near-IR pulses is 4 ps.

File name: Supplementary Movie 4

Description: Simulation of residual pulse formation in iDFG. The initial delay between the soliton and EO-comb pulses is swept from 5 ps to -5 ps. Assuming limited phase matching bandwidth, the walk-off between the two near-IR signals is 4 ps, the EO-comb is not compressed and is phase chirped. This case is close to actual experimental conditions, because, as noted in the main text, we lacked dispersion compensation units to properly compress both of the EO-combs.
