# Supplementary figures and images for: Architecture for microcomb-based GHz-mid-infrared dual-comb spectroscopy
Source: Nat Commun. 2021 Nov 12;12:6573. doi: 10.1038/s41467-021-26958-6 (PMC8589843; doi:10.1038/s41467-021-26958-6)

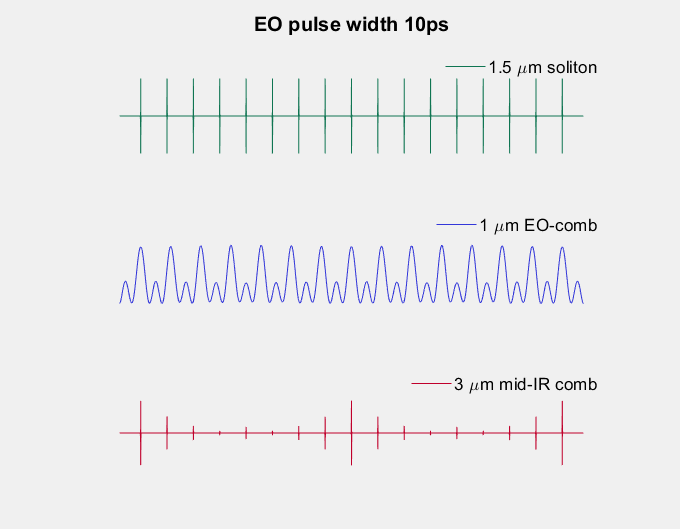

Supplement: Supplementary file 3 — Supplementary Movie 1 [file 41467_2021_26958_MOESM3_ESM.gif]

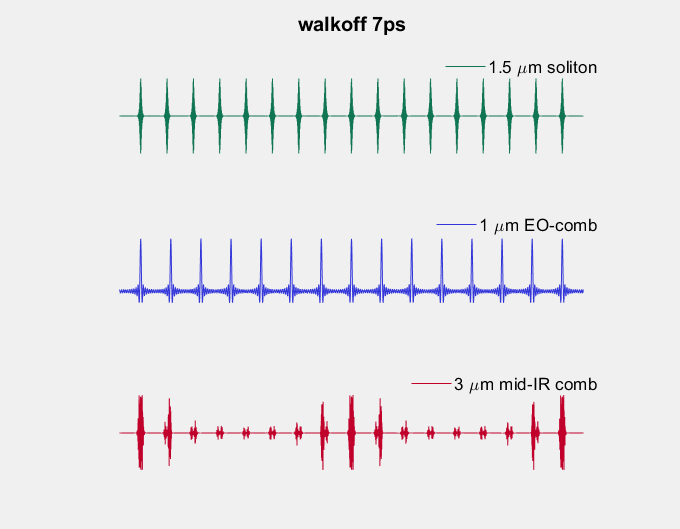

Supplement: Supplementary file 4 — Supplementary Movie 2 [file 41467_2021_26958_MOESM4_ESM.gif]

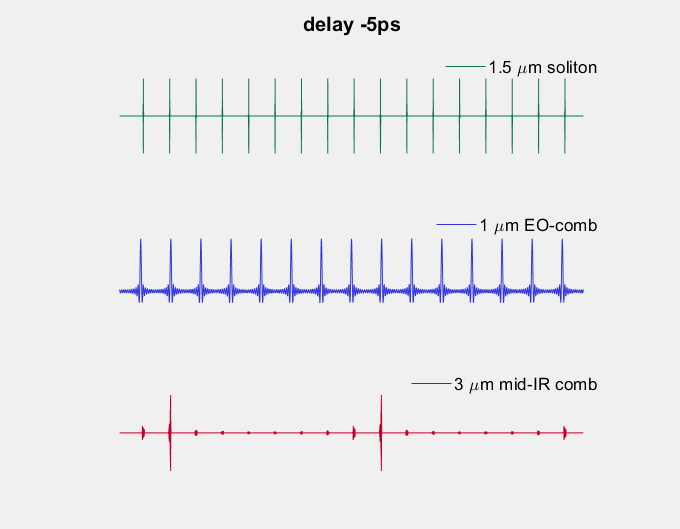

Supplement: Supplementary file 5 — Supplementary Movie 3 [file 41467_2021_26958_MOESM5_ESM.gif]

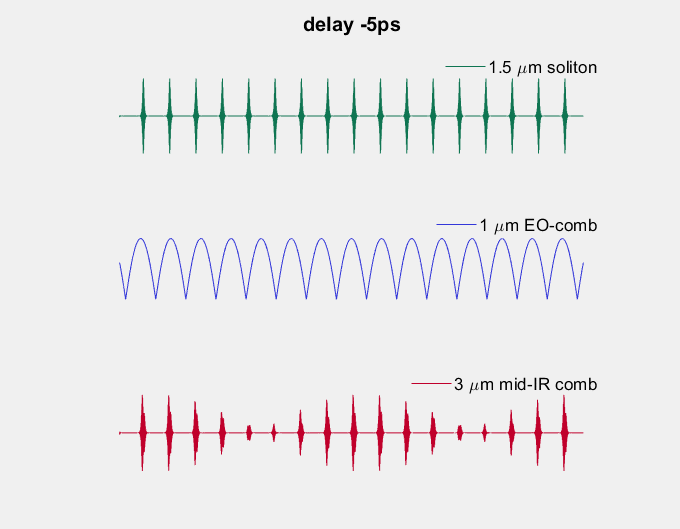

Supplement: Supplementary file 6 — Supplementary Movie 4 [file 41467_2021_26958_MOESM6_ESM.gif]
